# Detector-based spectral CT with a novel dual-layer technology: principles and applications

**DOI:** 10.1007/s13244-017-0571-4

**Published:** 2017-10-06

**Authors:** Negin Rassouli, Maryam Etesami, Amar Dhanantwari, Prabhakar Rajiah

**Affiliations:** 1 Department of Radiology, University Hospitals Cleveland Medical Center, Cleveland, OH USA; 2 Department of Radiology and Biomedical Imaging, Yale University School of Medicine, New Haven, CT USA; 3 Philips Healthcare, Cleveland, OH USA; 4 Cardiothoracic Imaging, Department of Radiology, UT Southwestern Medical Center, 5323 Harry Hines Boulevard, Dallas, TX 75390 USA

**Keywords:** CT, Dual-energy, Spectral, Contrast, Cardiac

## Abstract

Detector-based spectral computed tomography is a novel dual-energy CT technology that employs two layers of detectors to simultaneously collect low- and high-energy data in all patients using standard CT protocols. In addition to the conventional polyenergetic images created for each patient, projection-space decomposition is used to generate spectral basis images (photoelectric and Compton scatter) for creating multiple spectral images, including material decomposition (iodine-only, virtual non-contrast, effective atomic number) and virtual monoenergetic images, on-demand according to clinical need. These images are useful in multiple clinical applications, including- improving vascular contrast, improving lesion conspicuity, decreasing artefacts, material characterisation and reducing radiation dose. In this article, we discuss the principles of this novel technology and also illustrate the common clinical applications.

*Teaching points*

• *The top and bottom layers of dual-layer CT absorb low- and high-energy photons, respectively*.

• *Multiple spectral images are generated by projection-space decomposition*.

• *Spectral images can be generated in all patients scanned in this scanner*.

## Introduction

Conventional computed tomography (CT) scanners utilising a single X-ray spectrum are able to discriminate several tissues based on their attenuation (Hounsfield units, HU), but the overlap in attenuation of many tissues limits discrimination. Tissues have a unique distribution of attenuation values as a function of X-ray energy and spectral CT (dual-energy/multi-energy CT) can further distinguish them by exploiting their energy-dependent attenuation properties. Data collected simultaneously from two energy levels can be used to determine the Compton scatter and photoelectric components of x-ray attenuation which have different energy dependencies. These components provide additional information about tissue density and atomic number that can be exploited to separate tissues with similar attenuation in a conventional image. Spectral CT technologies so far have been source-based, including dual-source, rapid kilovoltage (kVp) switching and dual-spin technologies [1].

Detector-based spectral CT (SDCT; Philips Healthcare, Cleveland, OH, USA) offers a novel approach to dual-energy imaging where the spectral separation occurs at the level of the detectors. In this article, we describe data acquisition and image generation with SDCT and the clinical applications of this technology.

## Data acquisition hardware

**Fig. 1:**
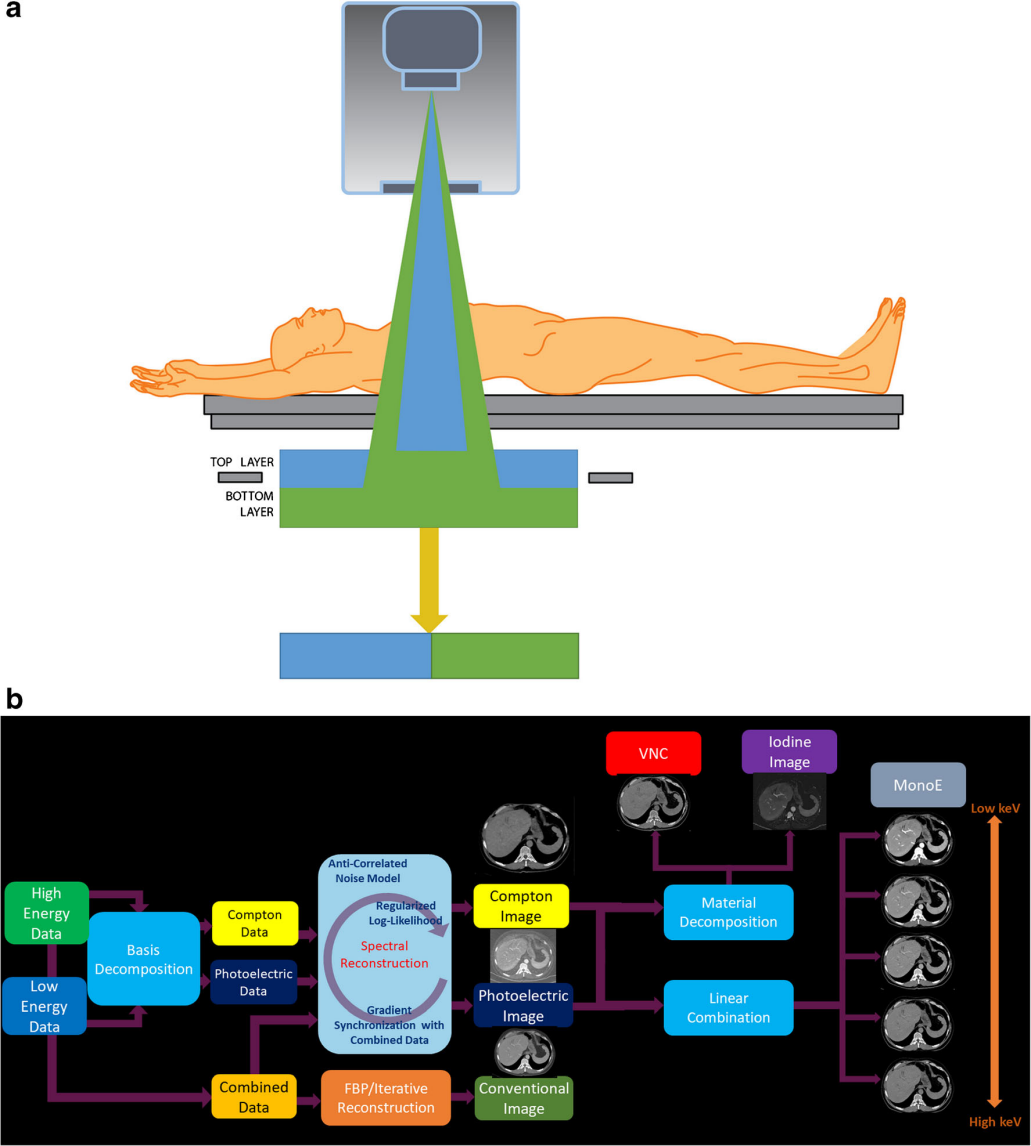
Spectral technology. (**a**) Illustration depicting the configuration of the dual-layer detector-based spectral CT. The top layer is made up of an yttrium-based garnet scintillator, which absorbs the low-energy photons and the bottom layer is made up of gadolinium-oxysulphide, which absorbs the high-energy photons. Attached to each layer, there is a thin front-illuminated photodiode (FIP), which converts the light photons to an electrical signal and an application-specific integrated circuit (ASIC) for analogue to digital conversion (not shown here). (**b**) Diagram showing the technique of image generation from the SDCT scanner. Data from the two layers is utilised to generate photoelectric and Compton scatter basis pairs. Linear combination gives virtual monoenergetic images, while material decomposition gives iodine-density, virtual non-contrast and effective atomic number-based images

SDCT utilises a single X-ray tube but has two layers of detectors; a top layer of an yttrium-based garnet scintillator and a bottom layer of gadolinium-oxysulphide (Fig. 1a). The top layer selectively absorbs low-energy photons while the high-energy photons penetrate this layer to reach the bottom layer where they are absorbed and converted into light. Attached to each layer, a  photodiode converts light into an analogue electrical signal and an application-specific integrated circuit converts it into digital signals. The X-ray tube has a 120-KW generator with tube voltages of 80–140 kVp. Scanner collimation is up to 64 × 0.625 mm yielding a maximum z-coverage of 40 mm. The fastest gantry rotation time is 270 milliseconds. [2, 3].

## Spectral decomposition

SDCT uses projection-space decomposition to solve for the basis components. X-ray attenuation of any material can be expressed as a linear combination of photoelectric and Compton scatter coefficients and, by extension, the attenuation coefficients at low energy and high energy can be expressed as the attenuation contributions from a predefined pair of basis materials [4]. More generally, the energy-dependent attenuation coefficient of a sample, *μ*(*E*), can be expressed as a linear combination of *n* basis functions *f*_*i*_ *(E)* and weighting constants α_*i*_ for *i = 1, 2,…,n*:

$$ \mu (E)={\upalpha}_1{f}_1(E)+{\upalpha}_2{f}_2(E)+\dots +{\upalpha}_n{f}_n(E) $$

Empirically, a pair of basis functions can be derived where the function *1/E* ^*3*^ closely approximates the photoelectric effect while *f*_*KN*_ *(E), defined as the Klien–Nishna function*, closely approximates Compton scattering:

$$ \mu (E)={\upalpha}_1\frac{1}{E^3}+{\upalpha}_2{f}_{KN}(E) $$

Solving for the weights *α*_*i*_ and *α*_*2*_ for each ray projection acquired at two different energies decomposes the raw data into photoelectric and Compton scatter basis components [5].

## Image generation

Several types of images are generated with SDCT, including conventional images and special spectral image types (Table 1).

**Table 1:** Types of images from the detector-based spectral CT scanner

Image type	Mechanism of generation	Clinical uses
Conventional (Polyenergetic/routine diagnostic)	Data from both layers considered as a single detector	Routine diagnostic use for all cases
Iodine density (iodine map)	Material decomposition with pixels representing iodine	Visualisation and quantification of iodine in vessels and organs of interest
Virtual non-contrast	Material decomposition and removal of iodine containing pixels	-Characterisation of lesions such as renal cysts/masses, adrenal nodules, lung nodules, etc. -Radiation dose saving by eliminating need for true non contrast
Uric acid pair	Material decomposition; depiction of pixels containing uric acid	Urinary calculus characterisation
Effective atomic number	Material decomposition; colour coding depending on atomic number	Tissue characterisation
Virtual monoenergetic	Linear combination of basis pair images (40–200 keV)	-Low monoenergetic: enhanced vascular contrast -High monoenergetic: decreased artefacts
Equivalent monoenergetic	Linear combination of basis pair, with attenuation values equivalent to conventional images	Higher image quality, with lower noise

### Conventional images (routine diagnostic/polyenergetic images) - 

These images are analogous to the images obtained from a single-energy scanner, and are utilised for routine diagnostic purposes. For every scan, the two detector layers are considered as a single detector and high- and low-energy data are combined. Filtered back projection or iterative reconstruction algorithms are used to reconstruct the combined raw data and create conventional images equivalent to those from a single-energy scanner. The image quality of these images have been shown comparable to images obtained from a single-energy scanner [6].

### Spectral images -

The basis pair raw data undergoes reconstruction to generate photoelectric and Compton scatter basis images, from which further additional material composition images are generated (Fig. 1b). Beam-hardening correction is inherent in this process, and image noise reduction is addressed in the reconstruction chain.

**Fig. 2:**
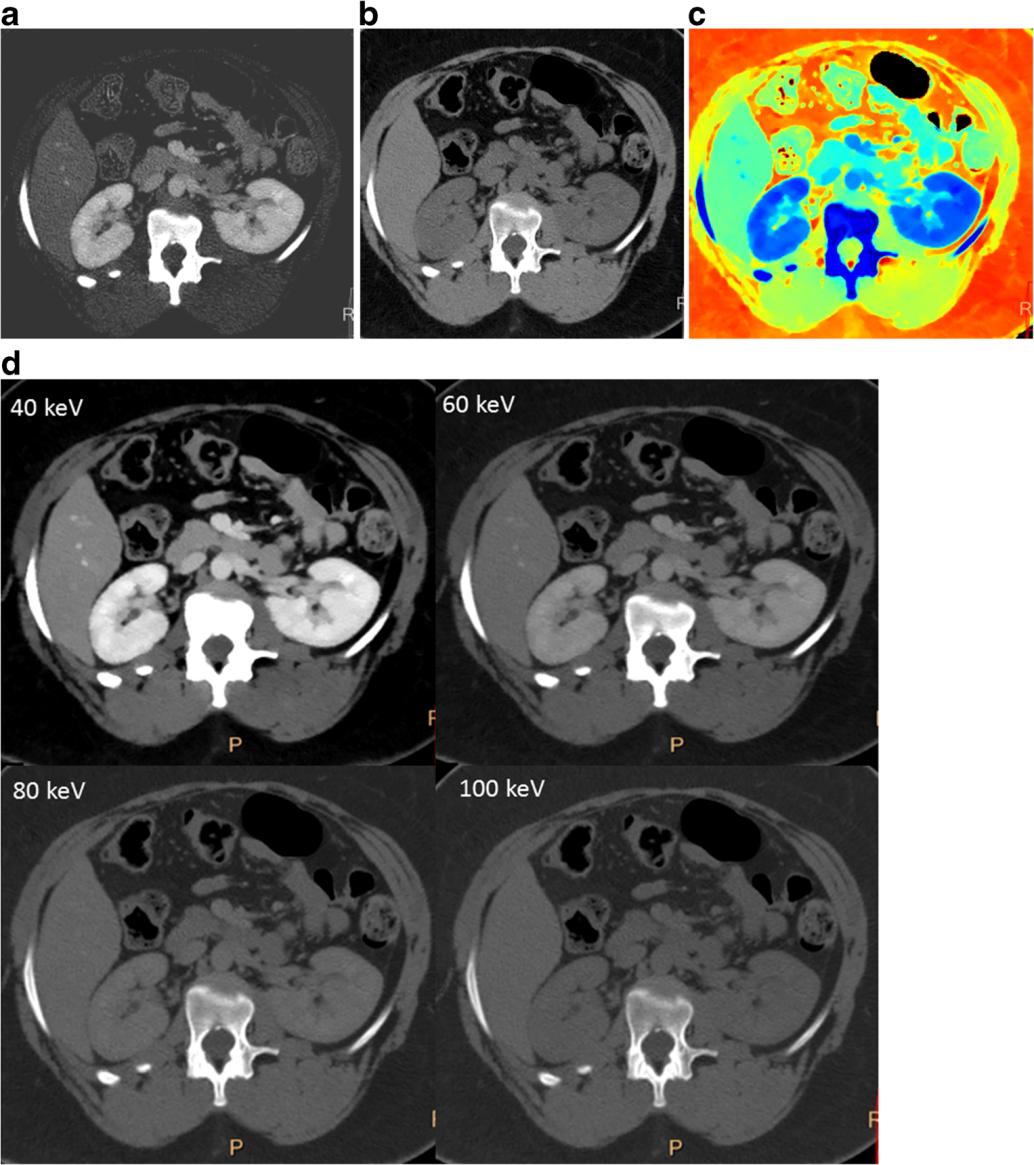
SDCT images. (**a**) Iodine-only image, highlighting tissues containing iodine. (**b**) Virtual non-contrast image, obtained after removal of pixels containing iodine. (**c**) Effective atomic number-based image, where the colour depends on the effective atomic number. (**d**) Virtual monoenergetic images at 40 keV, 60 keV, 80 keV and 100 keV. The contrast signal is highest in the 40-keV image, with the signal progressively decreasing at higher energy levels

- **Iodine density images-** Iodine-containing pixels are assigned values equal to the concentration of the iodine in each pixel, expressed in milligrammes/ml (Fig. 2a). Pixels containing no iodine appear dark. Iodine-only images permit quantification of iodine in vessels and organs.
- **Virtual non-contrast images-** Images where the contributions from iodine are removed, resulting in non-contrast-enhanced HU values of the tissue contained within the pixels (Fig. 2b). The resulting image mimics a true non-contrast-enhanced (pre-contrast) image.
- **Uric acid pair images-** A uric acid image displays only uric acid pixels with original HU values while all others appear dark. The uric acid-removed image is a complement to the uric acid image These images are utilised in the evaluation of urinary calculi composition and gout.
- **Z-effective images-** are colour-coded based on the effective atomic number of tissues (Fig. 2c). The coefficients of the photoelectric and Compton scatter components (α_1_ and α_2_, respectively) computed during the spectral decomposition process are functions of the spatial distribution of tissue [i.e., α_1_(*x*, *y*) and α_2_(*x*, *y*)]. The ratio of these coefficients is proportional to the effective atomic number, Z:

$$ \frac{\upalpha_1\left(x,y\right)}{\upalpha_2\left(x,y\right)}\propto \left({Z}^2=\frac{{\mathrm{Z}}^3}{\mathrm{Z}}\right) $$

Therefore, from this ratio we can estimate the effective atomic number as a function of (*x, y*) (2). The effective atomic number provides a higher level of discrimination than attenuation in HU because it depicts the material make-up of each pixel [4, 5].

- **Virtual monoenergetic images (VMI)** mimic images generated from application of a monoenergetic beam at a single kiloelectron voltage (keV) level. VMI are generated between 40 and 200 keV (Fig. 2d) by a linear combination of basis pair images. The attenuation, μ, at any energy can be determined from

$$ \int \mu \left(x,y;E\right) ds=\int {\upalpha}_1\left(x,y\right) ds\frac{1}{E^3}+\int {\upalpha}_2\left(x,y\right) ds{f}_{KN}(E) $$

VMI can better exploit the changes in tissue attenuation that occur with changing energy, particularly for tissues with high effective Z, compared to conventional images [4, 5].

- **Equivalent virtual monoenergetic images** mimic those generated by application of a monoenergetic beam at a single keV with attenuation values equivalent to conventional images but with lower artefacts and noise. For SDCT, the equivalent VMI levels are 70 keV for body, 66 keV for head and 64 keV for extremities [7].

## Advantages

A major advantage of SDCT is elimination of the requirement to prospectively select patients who need dual energy CT. All other currently available commercial solutions—dual-source CT, rapid tube potential switching and dual-spin technology—require selection of a special dual-energy protocol prior to scanning [8]. Spectral data are available for all patients imaged with SDCT without a change in clinical workflow, permitting evaluation of incidentally discovered findings and reduction of artifacts. The complete spatial and temporal alignment is ideal for cardiovascular studies and also allows projection-space decomposition with more accurate artefact correction and better noise removal in VMI compared to image space decomposition [9]. All radiation dose-reduction strategies available with conventional CT can be employed with SDCT. There are no restrictions related to field of view, gantry rotation time or cross-scatter.

## Limitations

Sufficient spectral separation requires a tube potential of at least 120 kVp. Therefore, radiation dose reduction for smaller patients relies on lowering the  tube current. Due to higher kVp, contrast is lowered in conventional images, but low-energy VMI may be helpful in such instances. The z coverage of the currently available scanner configuration is 4 cm, which makes some dynamic studies, such as myocardial perfusion, challenging.

## Clinical utility

There are several clinical applications of detector-based spectral CT, broadly divided into: (1) enhanced visualisation of contrast; (2) artefact reduction; (3) material decomposition; and (4) radiation dose reduction.

**Fig. 3:**
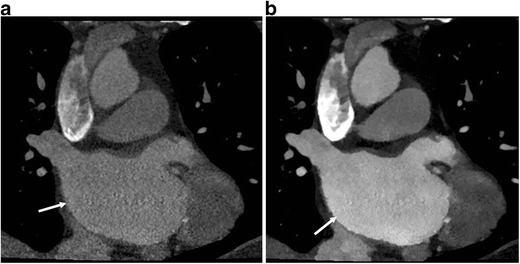
Salvage of a suboptimal vascular study. (**a**) Coronal 120-kVp routine diagnostic CT image in a patient being evaluated for radiofrequency ablation of pulmonary veins, shows poor contrast opacification of the vascular structures, particularly the left atrium (*arrow*), due to contrast extravasation during the scanning. (**b**) 45-keV virtual monoenergetic image (VMI) at the same level shows significant improvement in the contrast attenuation, especially in the left atrium (*arrow*). This obviates the need for an additional scan with another contrast bolus, saving radiation and contrast dose

**Fig. 4:**
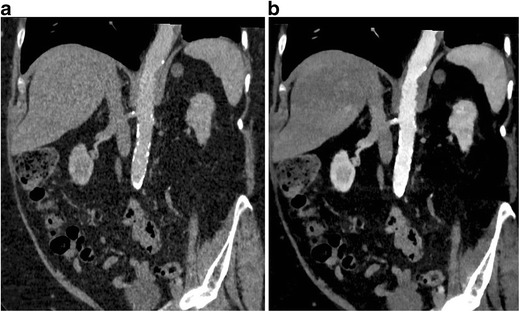
Low-contrast dose study. (**a**) Coronal 120-kVp routine diagnostic CTA image in a patient who received only 20 ml of intravenous contrast, shows poor contrast opacification of the abdominal aorta as well as parenchymal organs. (**b**) 40 keV VMI at the same level shows significant improvement of vascular contrast in the abdominal aorta. Thus, the low-energy VMI allows the use of low contrast dose, which is valuable in patients with severe renal dysfunction

**Fig. 5:**
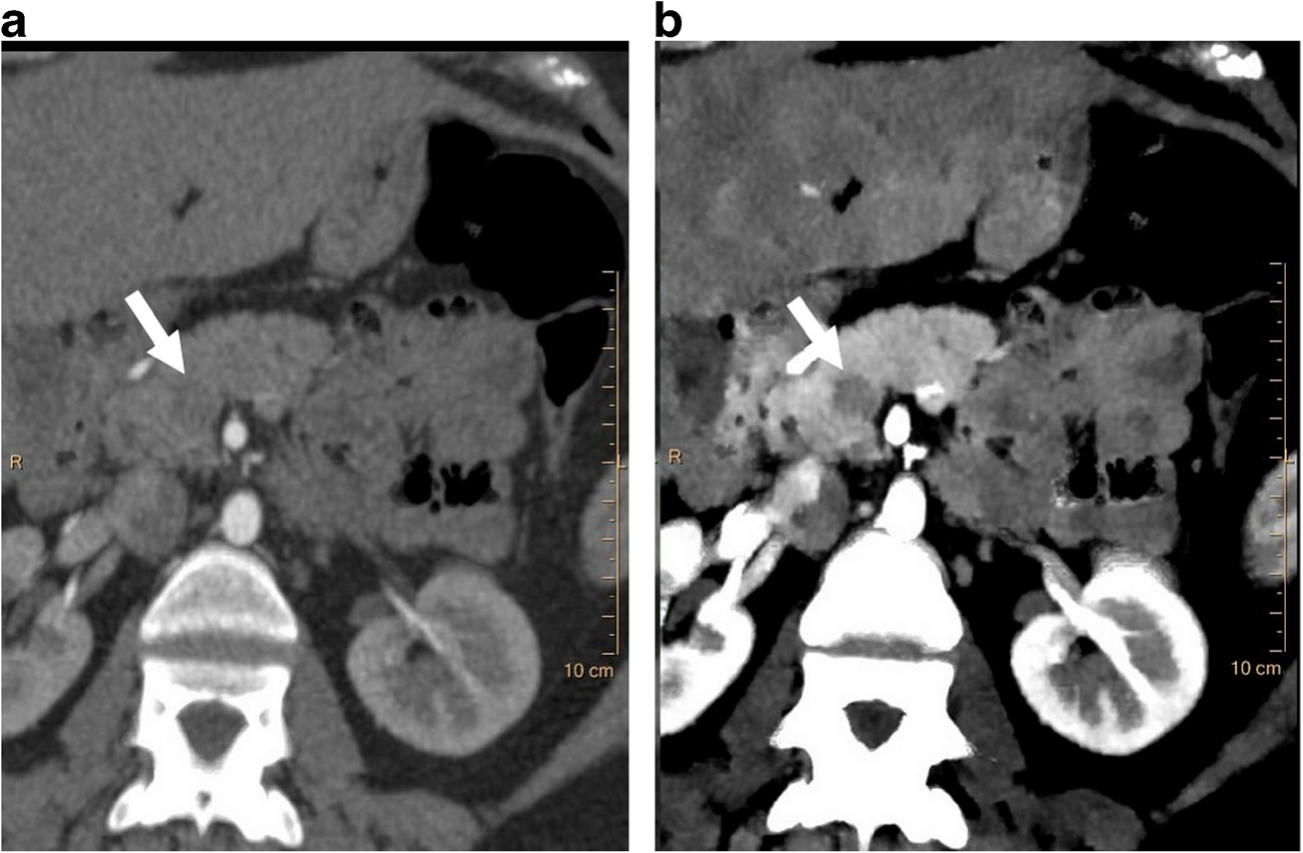
Improved lesion conspicuousness. (**a**) Axial 120-kVp routine diagnostic CT image through the upper abdomen shows a vague ill-defined hypoattenuating lesion in the pancreatic head (*arrow*). (**b**) 50-keV VMI at the same level shows excellent contrast opacification of the pancreas, with much improved conspicuity of the hypoattenuating pancreatic head lesion (*arrow*)
- **Artefact reduction**
  Several artefacts are commonly seen in CT, including metallic artefacts, beam hardening and calcium blooming [13]. Metals seen in strategic locations can limit the diagnostic confidence by obscuring vital structures. If the presence of metal is known ahead of time, metal reconstruction algorithms can be used. Retrospectively generated high-energy VMI from SDCT can be used to reduce/eliminate incidentally encountered metallic artefacts, particularly metals with low atomic number such as stainless steel and aluminium (Fig 6). Artefacts from high atomic number metals such as embolisation coils are improved with iodine maps. Beam hardening artefacts which are caused by the polyenergetic nature of the x-ray energy can also be minimised/eliminated by using the high-energy VMI (Fig 7). Calcium blooming is an important artefact in vascular imaging, which causes overestimation of stenosis and inappropriate classification of the lesion. This can also be reduced by using high-energy VMI [13]. This ability of the SDCT to minimise artefacts in critical areas, particularly retrospectively, may improve the diagnostic confidence.

- **Enhanced visualisation of vascular contrast**
  The attenuation of iodine is higher at lower energies due to the increased photoelectric attenuation at energies approaching the K-edge of iodine (33.2 keV). VMI reconstructions at lower energies (40–70 keV) can be used to enhance the visualisation of intravascular contrast, which can be clinically used in several settings [10, 11]. Suboptimal enhanced vascular studies are frequently encountered in imaging, either due to technical or patient factors. Usually, a suboptimal study requires a repeat study with additional bolus of contrast. However, with SDCT, due to the availability of low-energy VMIs on demand, suboptimal vascular studies can be salvaged, thus obviating the need for additional contrast injection or alternate imaging tests (Fig. 3). Although other currently available DECT scanners also have VMI capabilities, the dual-energy mode is not always switched on in these scanners and hence it may not always be available in a given patient. Vascular studies can be performed with low contrast dose, especially in patients with severe renal dysfunction and the contrast signal can be boosted by using VMI (Fig. 4). In addition, CT angiographic quality studies can be generated from routine contrast-enhanced CT scans, which may help in evaluation of incidentally seen vascular lesions. VMI, particularly at low energy or equivalent energy scans, can be used in improving the conspicuity of several lesions (Fig. 5). Hypervascular lesions may be more prominent at low-keV VMI due to improved iodine visualisation. Hypovascular lesions such as pancreatic adenocarcinomas are also well seen in low- or equivalent-keV VMI (Fig 5) [12].

**Fig. 6:**
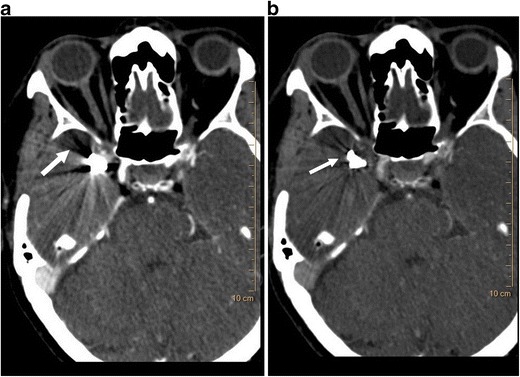
Metallic artefact reduction. (**a**) Axial 120-kVp routine diagnostic CT image through the head shows dense metallic opacity in the right temporal region (*arrow*). (**b**) 120-keV VMI at the same level shows significant improvement in the metallic artefact (*arrow*)

**Fig. 7:**
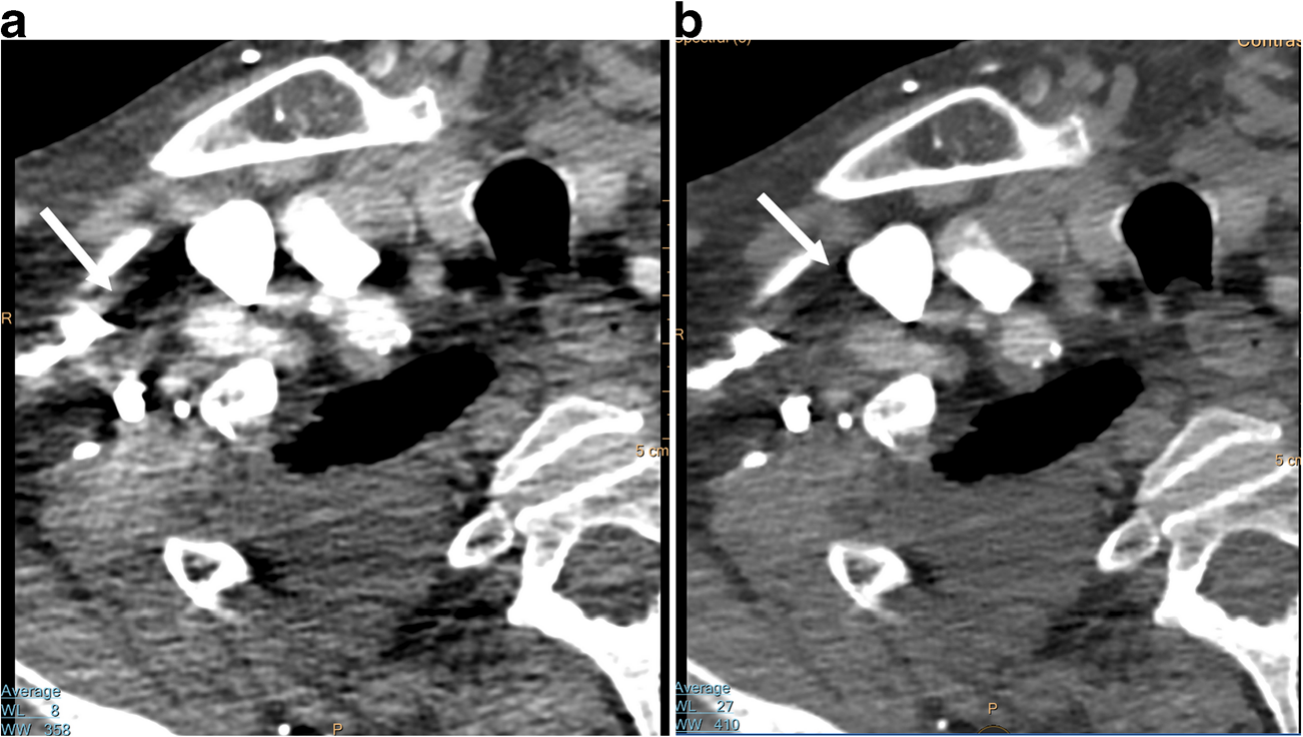
Beam hardening artefact reduction. (**a**) Axial 120-kVp routine diagnostic image at the level of the right axilla shows beam hardening artefact (*arrow*) originating from dense contrast within the right subclavian vein, which limits evaluation of adjacent structures. (**b**) 100-keV VMI at the same level shows near-total elimination of the beam hardening artefact (*arrow*)
- **Material decomposition**
  Z-effective images, iodine maps and calcium-uric acid images are currently used for the evaluation of urinary calculi, gout, lesion/plaque characterisation, perfusion of organs/tumour, tumour response to therapy, bone removal for CTA head, and virtual colonoscopy without bowel preparation. Increased utility may be possible with SDCT because of the opportunity to access spectral data for incidental findings. Characterisation of urinary calculi is important since management is different for uric acid and calcium calculi. Urinary calculi composition can be characterised by the z-effective images, and uric acid pair images (Fig 8) [14, 15]. Similarly, gout crystals can also be detected in joints. Iodine maps can be used to evaluate perfusion in several organs, including heart and lungs, the former in the diagnosis of myocardial ischemia and the latter in the evaluation of small pulmonary embolism (both acute and chronic) (Fig 9). Lesions involving the adrenal, kidney, liver and lung can be characterised with SDCT by a combination of VNC and iodine map, without the need for additional tests such as CT and MRI and possible additional radiation. For example, using VNC, the attenuation of an adrenal lesion from a contrast-enhanced image can be obtained and a lipid-rich adenoma can be diagnosed if attenuation is <10 HU [16]. A hyperattenuating renal lesion in contrast CT is likely to be a complicated cyst if there is high attenuation in VNC and no iodine uptake in the iodine map, and likely to be a solid tumour if there is no high attenuation in VNC, but there is iodine uptake (Fig 10). Some of these lesions usually require multi-phasic images for adequate characterisation.

**Fig. 8:**
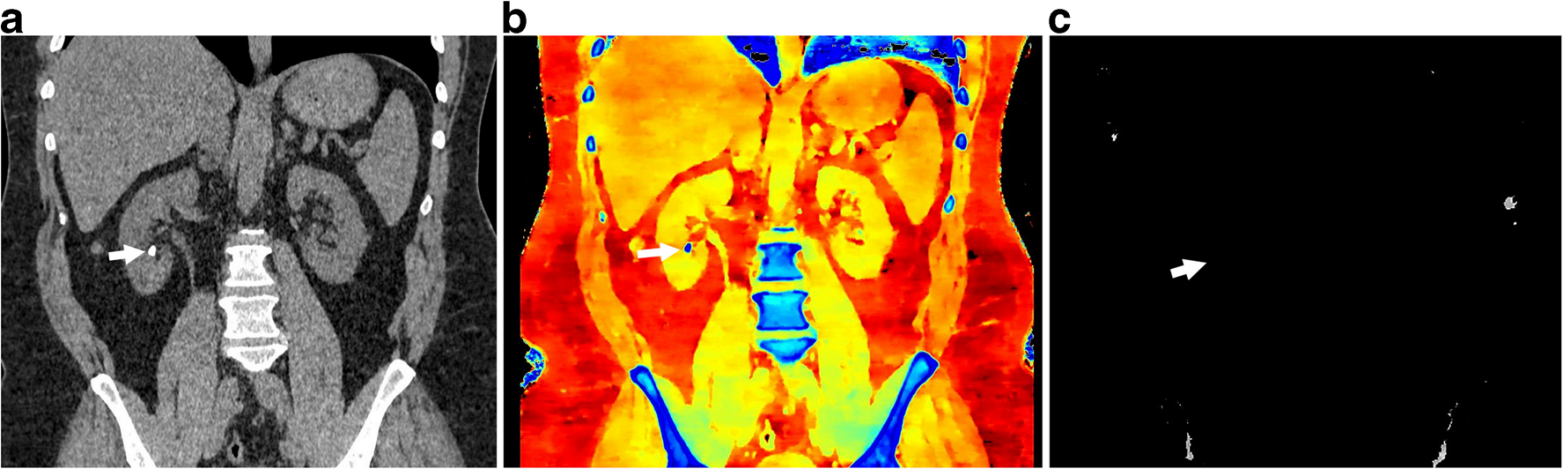
Urinary calculus composition. (**a**) Coronal 120-kVp routine diagnostic image in a patient with acute abdominal pain shows a 7-mm calculus in the inferior pole of the right kidney (*arrow*). (**b**) Effective atomic number-based reconstruction at the same level shows that the calculus has high atomic number (*arrow*) consistent with a calcium calculus. (**c**) Uric acid image shows no focal abnormality in the area corresponding to calculus (*arrow*), indicating that the calculus does not have uric acid

**Fig. 9:**
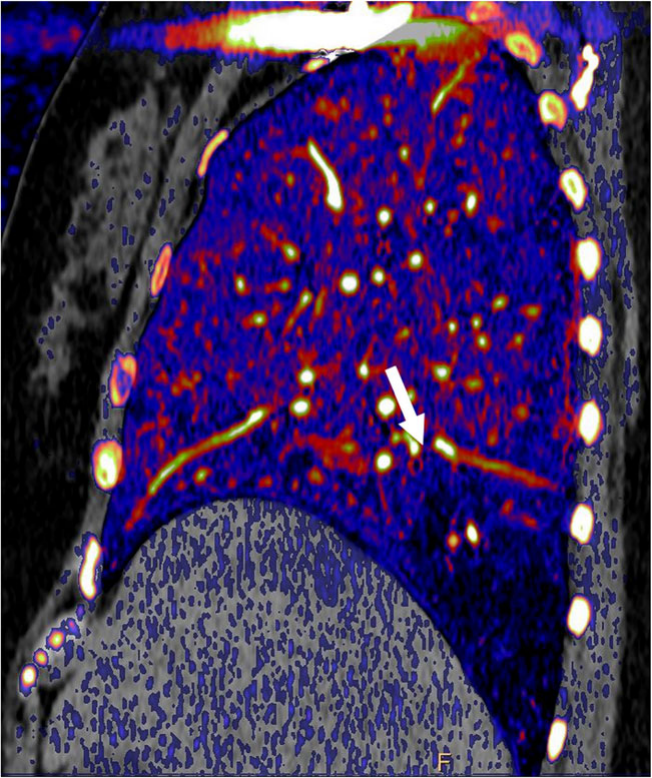
Perfusion imaging with iodine maps. Sagittal iodine overlay image in a patient with acute chest pain shows a wedge-shaped perfusion defect in the right lower lobe (*arrow*), which is consistent with an acute pulmonary embolism. CTA in this patient (not shown here) did not show the clot clearly

**Fig. 10:**
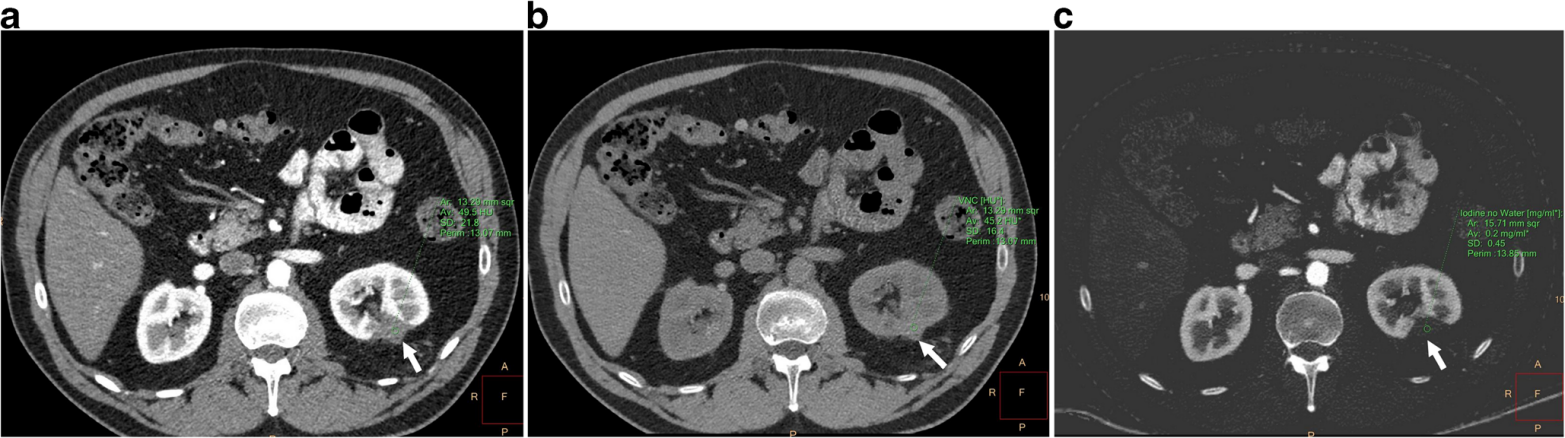
Lesion characterisation. (**a**) 120-kVp routine-diagnostic (conventional) CT image through the upper abdomen shows a well-defined hypoattenuating lesion in the left kidney (*arrow*), which has a mean attenuation of 49.5 HU, which is too high for a simple cyst. This can either be a complicated cyst or enhancing solid lesion. (**b**) VNC reconstruction at the same level shows that the attenuation of the lesion (*arrow*) is 45.2 HU, indicating that the high attenuation was inherent in the scan and not due to contrast administration (**c**) Iodine map at the same level confirms the above, with an insignificant (0.2 mg/ml) iodine uptake in the lesion (*arrow*). This constellation of findings helps in diagnosing this as a complicated renal cyst than an enhancing solid mass
- **Radiation dose reduction**
  The attenuation values of the VNC images from SDCT have been shown to be similar to true unenhanced images in all tissues except for subcutaneous fat [17]. Hence, VNC images may eliminate the need for true non-contrast scans in multi-phasic studies, thus reducing radiation dose (Fig. 11). Radiation dose savings of up to 40% has been shown by eliminating the true non-contrast phase and up to 64% by eliminating the arterial phase [18, 19]. The ability to salvage suboptimal enhanced studies and characterise incidental findings (e.g., adrenal, renal lesions) may also obviate the need for an additional CT scan, thereby reducing radiation dose.

**Fig. 11:**
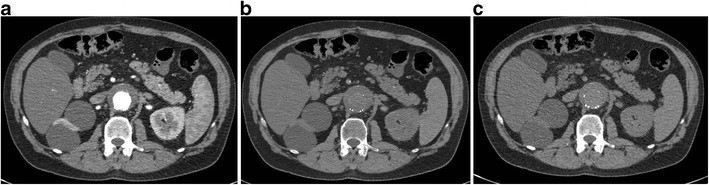
Virtual non-contrast. (**a**) Axial 120-kVp routine diagnostic image at the level of kidneys shows a contrast-filled stent graft lumen within the proximal abdominal aorta. (**b**) Virtual non-contrast reconstruction at the same level has removed the pixels which contained iodine. (**c**) Compare this with the true non-contrast image obtained at the same level, which shows similar appearance and attenuation values as the virtual non-contrast. In multi-phasic studies, especially in vascular indications, the true non-contrast phase can be excluded, resulting in significant radiation dose savings

## Conclusion

SDCT is a unique technology utilising dual-layer detectors which provides spectral data on demand for all patients. There are several applications of this technology in several organ systems. The major applications of this technology are to improve the vascular contrast using low-energy VMI images; decrease several artefacts using high-energy VMI images; material composition of several tissues and lesions using an iodine map, VNC and effective-z images; and radiation dose savings using VNC images instead of true non-contrast images as well as saving additional CT examinations for lesion characterisation.
